# The Opioid Crisis: How to Lessen the Burden on Emergency Departments by At-risk Populations

**DOI:** 10.7759/cureus.11498

**Published:** 2020-11-16

**Authors:** Sapan Patel, Asad Sheikh, Natasha Nazir, Shannon Monro, Ammaar Anwar

**Affiliations:** 1 Epidemiology, Touro College of Osteopathic Medicine, Middletown, USA; 2 Medicine, New York Institute of Technology College of Osteopathic Medicine, Old Westbury, USA

**Keywords:** opioid abuse, emergency departments, mental health services, community, substance abuse and mental health services administration, public health service

## Abstract

Background

The opioid crisis in the United States of America has been worsening due to previous sharp increases in opioid prescriptions and a lack of resources available to those affected. Emergency departments (ED) across the nation have been exhausted with a constant influx of patients related to opioid-related issues. Because of limited resources, it is crucial to efficiently distribute rehabilitation and mental wellness efforts amongst those most susceptible to opioid abuse. By identifying common environments and characteristics of the population presenting to ED’s for opioid-related issues, we can (1) lessen the economic burden on the healthcare system while (2) increasing the rate of successful treatment for those affected by opioid addiction.

Methods

Data was obtained regarding ED visits for opioid-related issues at the level of all 50 states from the Healthcare Cost and Utilization Project's (HCUP) State Emergency Department Databases (SEDD) and nationally from the Nationwide Emergency Department Sample (NEDS). Rates of ED admissions for opioid-related issues were statistically analyzed to identify characteristics of the population that are most at risk for presenting to the ED for opioid-related issues.

Results

Statistical analysis showed residents of Large Metropolitan areas (M=351.94, p=0.022, CI±42.89), those earning incomes below the 25th percentile (M=359.14, p=0.008, CI ±61.39), and 25-44-year-old population (M=456.71, p=0.001, CI±27.01) to be the most likely subset of the population to report to the ED for opioid-use issues. Conversely, those earning incomes above the 75th percentile were significantly less likely to utilize ED’s for opioid-related issues (M=143.10, p=0.026, CI±0.026).

Conclusion

Results demonstrated that patients between the ages of 25 and 44 were more likely to develop opioid use disorders. This provides an opportunity to educate this population via opioid education centers. Additionally, residents of Large Metropolitan areas would benefit from naloxone distribution centers. Lastly, higher income levels appear to be related to a decrease in ED presentation for opioid abuse. This provides evidence for medication-assisted therapy (MAT) to be provided by low-income insurance plans.

## Introduction

One of the most significant public health challenges that the world faces today is substance use disorder. In 2017, drug overdoses caused 585,348 fatalities globally, while $200-250 billion USD was spent on treatment-related expenses in just one year [1,2]. Drug abuse puts an immense burden on healthcare systems worldwide. The particular interest of this study is the draining effects that opioid abuse has on ED’s in the United States of America. This may also serve as a model to demonstrate the burdens that substance use disorders place on other developed countries. 

Paradoxically, a large contributing factor to the increase in opioid abuse is the liberal prescription of opioids. While there have been increasing rates of opioid misuse, efforts for risk reduction have failed to present at the same rate. Reports from a study analyzing ED activity found a 29.5% increase in opioid overdoses in 45 states from 2016 to 2017 and a 69.7% increase in opioid overdoses in certain regions of the United States [3,4]. Sixteen states with large cities experienced an opioid overdose increase of 54.1% [5]. 

Drug overdoses are now the leading cause of death for those under 50 years of age. Substance abuse costs the United States about $78 billion annually in lost productivity, criminal justice, and healthcare expenses [5]. In addition to the economic burden that the opioid epidemic has placed on the American economy, it has vast implications on the healthcare system. Individuals that struggle with opioid misuse and abuse consume high levels of healthcare resources, including outpatient, emergency, and inpatient stays. In 2014, 147,654 ED visits were due to opioid overdoses alone [6]. Examples of complications that could arise from the opioid epidemic include higher medical expenditures, a decrease in available healthcare workers to care for other patients, fewer hospital beds, extended waiting periods, increased workload for healthcare providers, and avoidable stress on ED’s. 

One effort to mitigate the recent rise in opioid abuse is the implementation of prescription drug monitoring programs (PDMP) or state-level electronic databases that are regulated and required by providers. Since 2018, 41 states require providers to use the PDMP [6].

This study aims to compare the rate of ED visits by opioid users in regions across the United States. Parameters, such as age and income level were also analyzed. By identifying populations at risk for opioid abuse, healthcare providers (HCP), public health officials, and policymakers can allocate resources more efficiently. By identifying common environments and characteristics of the population presenting to ED’s for opioid-related issues, we can lessen the economic burden on the healthcare system while increasing the rate of treatment for those affected by opioid addiction.

## Materials and methods

Unit of analysis

Emergency Department (ED) visits include ED encounters for patients defined by selected the 10th revision of the International Classification of Disease (ICD-10) codes from the F11 Series (“Opioid-related disorders”) and select codes from the T40 Series (“Poisoning by, adverse effect of and underdosing of narcotics”). T40 Series specified for “Opium, Heroin, Other Opioids, Methadone”, “Other synthetic narcotics”, “Unspecified narcotics”, “Other narcotics”. “Rates” were calculated with the numerator being the number of hospital discharges of a patient admitted for at least one of the aforementioned ICD-10, and the denominator being the United States resident population divided by 100,000.

Data obtained for each state are from the Healthcare Cost and Utilization Project's (HCUP) State Emergency Department Database (SEDD) [7]. Data from the American Hospital Association (AHA) and Survey of Hospitals and the Trauma Information Exchange Program (TIEP) are used to weight for the 5% of EDs or 2% of ED visits that are not included in SEDD.

Location

Counties categorized patient location with a 6-leveled classification developed by the National Center for Health Statistics (NCHS). “Large central metropolitan” locations are defined as counties in metropolitan statistical areas (MSAs) with a 1+ million population that contains a minimum of 250,000 inhabitants. Counties that did not have a principal city of at least 250,000 inhabitants, but had a population of 1+ million were determined to be suburban or “Large Fringe Metropolitan.” Furthermore, counties in MSA’s with populations of 250,000-999,999 and <250,000 were characterized as “Medium Metropolitan” and “Small Metropolitan,” respectively. Lastly, “Rural” locations were characterized by patients located in micropolitan statistical areas (Micropolitan) and other non-metropolitan counties (Noncore) [7].

Income level

Community-level income was based on the median household income of the patient's ZIP code of residence. Quartiles were defined so that the total U.S. population was evenly distributed across the four groups.

Age

Age refers to the age of the patient at admission. Discharges or visits missing age are excluded from results reported by age.

## Results

Residents of Large Central Metropolitan areas (M=351.94, p=0.022, CI±42.98) reported to ED’s for opioid-use more often than the population as a whole. Although statistically insignificant, Rural and Small Metropolitan areas showed a correlation between higher rates of ED admissions and opioid-related issues. Those earning incomes below the 25th percentile (M=359.14, p=0.008, CI±61.39) were more likely to be admitted to the ED, t = 4.23, p < 0.05, while those earning in the top quartile of the population (M=143.10, p=0.026, CI±12.56) were significantly less likely to report to ED for opioid-use, t = -5.33, p < 0.05. 25 - 44-year-olds (M=456.71, p=0.001, CI±27.01) are significantly more likely to be admitted to the ED for opioid-use compared to the rest of the population, t = 7.48, p < 0.05. The 25-44-year-old age cohort showed the most significance as a predictor for the usage of the ED for opioid-related issues leaving remaining age cohorts as remarkably insignificant contributors to this issue (Table 1).

**Table 1:** Emergency department visits due to opioid-related use by location, income level, and age *“Rates” were calculated with the numerator being the number of hospital discharges of a patient admitted for and ICD-10 code related to opioid-substance-use-disorders, and the denominator being the United States resident population divided by 100,000. ** Statistical analysis was conducted using two-sample equal variance (homoscedastic) T-tests and 95% confidence intervals/margins of error. ICD-10: 10th revision of the International Classification of Disease.

Location	Mean	P-Value	Margin of Error
Large Central Metropolitan	351.94***	0.022	42.89
Large Fringe Metropolitan	249.47	0.873	31.79
Medium Metropolitan	261.00	0.709	30.17
Small Metropolitan	206.53	0.058	25.21
Rural	222.71	0.122	25.77
Income Level (Percentile)	Mean	P-Value	Margin of Error
0-25%	359.14***	0.008	61.39
26-50%	260.90	0.412	34.34
51-75%	206.64	0.096	22.64
s76-100%	143.10***	0.026	12.56
Age (Years)	Mean	P-Value	Margin of Error
1-24	102.11	0.124	10.64
25-44	456.71***	0.001	27.01
45-64	252.05	0.351	28.82
65+	110.74	0.096	8.97

## Discussion

Overview

Our statistical analysis of locations, income levels, and ages of ED visits for opioid-related use identifies subpopulations of patients that are significantly affected by the opioid crisis and that utilize ED department resources. These demographics and parameters show wherein the opioid crisis the most vulnerable and affected populations lie. ED’s in “Large Central Metropolitan” areas were found to be more stressed with opioid-related visits, while patients between the ages of 25 - 44 years old accounted for the highest cohort of opioid-related ED visits. People with income levels below the 25th percentile were more likely to report to the ED for opioid-related issues. Conversely, findings showed that people with income levels above the 75th percentile were less likely to present to the ED for opioid-related illnesses when compared to the rest of the population. These findings provide potential information to help HCPs, public health officials, and policymakers efficiently combat the crisis, and may also offer preventative solutions and provide the opportunities for a more focused approach to lessen the burden on the healthcare system.

Location

Our findings showed large central metropolitan areas had more significant ED admission rates related to opioid use disorder than in small metropolitan and rural areas, which were replicated from results in the 2016 National Survey on Drug Use and Health [8]. While federal and other national initiatives have been targeting rural areas for improvements in prevention, harm reduction, and treatment, large metropolitan areas face slightly different problems in demand rather than supply. Firstly, a 2015 article analyzing a National Survey on Drug Use and Health showed that 5.9% of urban adults reported non-medical use of prescription opioids, compared to 4.9% of rural adults [9]. Metropolitan communities, noted for their economic success, often masks greater wealth inequality and housing instability compared to other communities. A study from the U.S. Department of Housing and Urban Development’s Office of Policy Development and Research showed 71% of homeless individuals were located in central cities compared to 21% and 9% of homeless people located in suburban and rural areas, respectively. Additionally, findings showed homeless individuals in central cities had median incomes of $250 compared to homeless in locations such as suburban areas and rural areas, with median incomes of $395 and $475, respectively [10]. With a lack of housing available in large metropolitan communities, homeless individuals are left with little resources when addressing non-urgent medical care and failure to prevent illnesses and injuries, leading to the overcrowding of ED’s. In a survey of 2,578 homeless and marginally housed individuals, it was noted that 54.5% of all ED visits were from 7.9% of the sample [11]. The more significant rates of ED admissions in “Large Central Metropolitan” areas can, therefore, be partially attributed to the compounding effect of inadequate mental health resources available for financially scarce individuals and housing scarcity which together, leads to an increase in substance use disorders and the recurrent seeking of ED resources for shelter.

Income level

Health coverage from job-based plans, health insurance marketplace plans, and individual plans outside of the marketplace are reserved for the financially stable. Those in the 25th percentile of income either receive subsidized health coverage from Medicaid and the Children’s Health Insurance Program (CHIP) or left uninsured. The 25th percentile of income included households earning less than $30,725.01; this is approximately equal to the Federal Poverty Level (FPL) for households with a family of 5, or $30,680 [12-13]. While states that have expanded Medicaid coverage can provide health insurance to a household below 133% of the federal poverty level, populations in states without expanded Medicaid often have barring restrictions for qualifying for income-based Medicaid. Additionally, a rising 8.5%, or 27.5 million people, did not have any health insurance during 2018 [13]. By expanding healthcare initiatives, resources would be more readily available to those that are in need and would serve as a convenient avenue to lessen public health issues. 

Age

This study showed that 25-44 years olds are significantly more likely to be admitted to the ED for opioid use than the rest of the population. This age cohort is more susceptible to synthetic opioid use, loosely regulated opioid prescribing practices, and financial burdens. More specifically, the increase in fentanyl use from the years 2013-2017 led to increased addiction and adverse outcomes such as abuse and death [14]. Millennials also increased their use of heroin since 2013, while 75% of heroin addicts reported their first introduction to opioids was via prescription painkillers [15-17]. Heroin is sought out by opioid addicts and users because it is easier to obtain than prescription opioids because of their higher prices and restrictions. Conclusively, the significant rates of attendance to the ED for opioid-related issues by 25-44-year-olds could possibly be attributed to financial burdens, such as student loan debt, high costs of housing, raising young children, establishing careers, and lack of health insurance. These factors can lead to feelings of stress, anxiety, and hopelessness. Opioids can be sought by this age group to help alleviate these feelings.

Prevention and education

Education and counseling on opioids can be used to help combat the crisis. Education can be provided on the adverse effects of opioids, along with counseling for users and addicts to aid them in recovering. By targeting this age group, it would be reasonable to forecast a downward trend in opioid-related ED visits. Needle exchange programs, mostly in urban settings, can be increased in areas where populations of 25-44-year-olds are abundant. Education for HCPs should be present before opioids are prescribed, alongside stricter guidelines to lead to fewer unnecessary opioid prescriptions and consequent substance use disorders.

## Conclusions

This study, utilizing data from SEDD and NEDS databases, showed that patients between the ages of 25 and 44 are more likely to develop opioid use disorders. This provides possible evidence for the benefits of educating this population via opioid education centers. In addition, residents of Large Metropolitan areas would benefit from naloxone distribution centers. Lastly, income levels show to be a significant indicator of opioid abuse, which could provide evidence for medication-assisted therapy (MAT) to be encompassed by low-income insurance providers. Education and counseling on opioid use can be used to help combat the crisis. By defining and targeting at-risk subpopulations, resources can be more efficiently distributed to aid those most affected and attenuate the opioid crisis.
